# Size-dependent guest-memory switching of the flexible and robust adsorption characteristics of layered metal-organic frameworks

**DOI:** 10.1126/sciadv.adr1387

**Published:** 2024-12-06

**Authors:** Satoshi Watanabe, Shotaro Hiraide, Homare Arima, Akiko Fukuta, Miyuki Mori, Hideki Tanaka, Minoru T. Miyahara

**Affiliations:** ^1^ Department of Chemical Engineering, Kyoto University, Katsura, Nishikyo, Kyoto 615-8510, Japan.; ^2^ Institute for Aqua Regeneration (ARG), Shinshu University, 4-17-1 Wakasato, Nagano 380-8553, Japan.

## Abstract

Flexible-robust metal-organic frameworks (MOFs), which exhibit unique hybrid nature comprising both flexible and rigid framework characteristics, exhibit high potential for hydrocarbon separations. However, no clear guidelines have been established to regulate their hybrid characteristics owing to limited understanding of their adsorption mechanism. This study investigates the effects of the particle size of a flexible-robust MOF on its adsorption and structural transition behaviors. The robust nature originates from the structural transition of a metastable guest-free structure, while its flexible nature arises from another guest-free structure. The type of guest-free structure is predominantly determined by the particle size; particles below the critical size are trapped in the metastable guest-free structure. Notably, the critical size varies with the type of guest molecule to be removed; consequently, the difference in critical size results in guest-memory characteristics, enabling guest-free structure switching. These results underscore the importance of controlling the particle size to fine-tune hybrid adsorption characteristics of flexible-robust MOFs.

## Teaser

Particle size control of flexible-robust MOFs enables the switching of flexibility and rigidity of their framework structure.

## INTRODUCTION

The adsorption separation of gases, with energy-saving, high-efficiency, and low-cost characteristics, is a key technology that can facilitate the development of a sustainable society (*1*–*4*). Several types of adsorption processes—including pressure-, vacuum-, and temperature-swing adsorption—are used for treating different feed gases with widely varying volumes and compositions. To manufacture adsorption systems with a high separation efficiency that can capture specific target molecules, the tailored design and control of the adsorbent structure and properties are crucial. Metal-organic frameworks (MOFs), which are porous crystalline materials comprising metal ions and organic ligands, exhibit high potential for gas adsorption because the vast variety of metal ion-ligand combinations facilitate the formation of adsorbents with a controlled pore size, surface area, and molecule adsorption affinity (*5*, *6*).

MOFs are generally classified as rigid or flexible. Rigid MOFs show permanent porosity, high surface areas, and large pore volumes, typically yielding type I isotherms, while flexible MOFs undergo a crystal-structure transformation from a closed/narrow pore phase to a large pore phase in response to external stimuli (*7*, *8*), resulting in the stepwise uptake of guest molecules by gate adsorption (*9*). Thus, flexible MOFs exhibit high working capacities in separation processes (*10*, *11*) and molecular recognition abilities in sensing applications (*12*). Flexible-robust–type MOFs, which were originally proposed by Li *et al.* in 2017 (*13*), exhibit unique hybrid properties characterized by multistep adsorption isotherms, which comprise a type I profile in the low-pressure region and a subsequent stepwise increase at a certain pressure (*14*–*17*). Owing to their hybrid nature, flexible-robust MOFs exhibit high potential for the challenging separations of several hydrocarbon mixtures such as C_2_H_2_/C_2_H_4_, C_2_H_4_/C_2_H_6_, and C_3_H_4_/C_3_H_6_ (*13*, *18*–*21*). Fine-tuning the hybrid characteristics of flexible-robust MOFs would improve their adsorption and separation performances substantially. However, unlike the robust literature on MOFs with rigid or flexible properties, very little information is available on the fundamentals of flexible-robust MOFs and the mechanism of their adsorption and structural transition behaviors; therefore, the guidelines for controlling the hybrid characteristics of flexible-robust MOFs remain ambiguous. Similar to flexible MOFs, which exhibit particle-size–dependent characteristics such as adsorption kinetics (*22*), framework flexibility (*23*, *24*), and hysteretic behavior (*25*), the particle size of flexible-robust MOFs is a possible physical factor that affects the structural flexibility of these systems without changing their material composition. However, to the best of our knowledge, the influence of the particle size of flexible-robust MOFs on their properties has not been investigated to date.

This study investigates the impact of the particle size of a flexible-robust MOF on its adsorption and structural-transition behavior. The elastic layer-structured MOF-12 {ELM-12 [Cu(OTf)_2_(bpy)_2_)]; trifluoromethanesulfonate (OTf); 4,4′-bipyridine (bpy)}, which was the first-reported MOF to exhibit a double-step isotherm (*14*), was used as the model flexible-robust MOF in this study. ELM-12 shows a stacked structure of two-dimensional (2D) tetragonal grid sheets comprising Cu ions and bpy molecules and intersheet pillars comprising OTf anions that bind with Cu ions and exhibits unique adsorption behaviors toward toxic gases such as SO_2_ and N_2_O (*26*, *27*). For this study, monodisperse ELM-12 particles were downsized. The typical synthesis of ELM-12 involves carefully layering a bpy solution onto a Cu(OTf)_2_ solution to promote an interfacial chemical reaction between the two solutions. However, as the interfacial reaction is a slow process, it facilitates the synthesis of relatively large crystals (in the order of 10^2^ μm) with a wide size distribution, which are unsuitable for this study. Therefore, to synthesize monodisperse ELM-12 particles of different sizes, a bpy solution was intensively mixed with a Cu(OTf)_2_ solution using a microreactor to form a uniform reaction solution; this mixing induced instantaneous nucleation and the subsequent growth of ELM-12 particles. Microreactors are promising reaction tools because of their excellent mixing ability (*28*, *29*), as demonstrated in our previous studies in which we applied a central collision-type microreactor to particle synthesis (*30*) and successfully synthesized different types of particles such as monodispersed MOF particles (*31*, *32*), metallic nanoparticles (*33*), and nanoshells (*34*, *35*).

In this study, the particle size dependence of the structural variations and adsorption behavior of ELM-12 were explored. ELM-12 particles with different sizes were synthesized by using a central collision-type microreactor. The N_2_ adsorption isotherms and structure of these particles were investigated to elucidate the origins of the robust and flexible characteristics specific to ELM-12 particles. Through the particle size–dependent behavioral analyses, we successfully clarified that the initial type I–like uptake by ELM-12 particles is caused not by a “rigid” nature of the framework, which was the originally proposed concept (*13*), but by its “extremely flexible” nature specific to a metastable guest-free structure that responds to very low pressures, while the stepwise uptake is due to the formation of another guest-free structure. Furthermore, on the basis of the understanding on the flexibility difference between guest-free structures of ELM-12, adsorption isotherms of H_2_ and D_2_ were investigated as a potential application of ELM-12 to an isotope separation. This study provides a guideline for regulating the hybrid adsorption properties of flexible-robust MOFs that can facilitate the development of high-efficiency adsorption-based separation processes.

## RESULTS

### Synthesis of ELM-12 particles

**Fig. 1.**
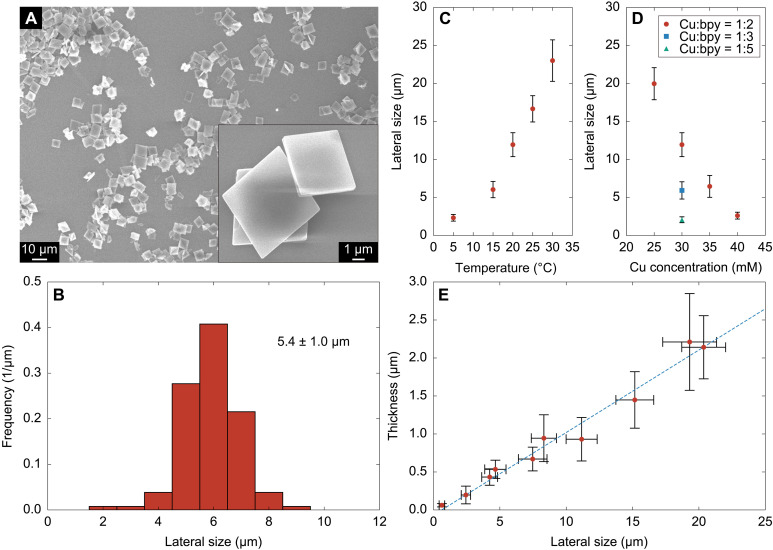
ELM-12 particles produced by the microreactor-based synthesis. (**A**) Representative SEM image and (**B**) lateral size distribution of ELM-12 particles synthesized with Cu and bpy concentrations of 30 and 60 mM. The dependence of the average lateral size of ELM-12 particles on (**C**) the reaction temperature and (**D**) Cu concentration. (**E**) Linear relationship between lateral size and thickness. Error bars indicate standard deviation of data.

ELM-12 particles were synthesized by mixing an aqueous solution of Cu(OTf)_2_ with an ethanol (EtOH) solution of bpy in a central collision-type microreactor with an excellent mixing ability. Figure 1 (A and B) shows the particles generated by the typical microreactor-based synthesis. These particles are well-dispersed and plate-like rhombic in shape (Fig. 1A) with a lateral size of 5.4 ± 1.0 μm (Fig. 1B). Intensive mixing by the microreactor enabled the synthesis of particles with a narrow size distribution; semi-batch–type mixing with a weaker mixing ability produced particles with a wider size distribution (fig. S1). The lateral size of particles increased with the temperature and decreased with the Cu concentration and the Cu-to-bpy ratio within 2 to 23 μm (Fig. 1, C and D), possibly because higher supersaturation conditions at lower temperatures, higher Cu concentrations, and larger Cu-to-bpy ratios produced a larger number of smaller nuclei, resulting in the formation of smaller-sized particles [scanning electron microscopy (SEM) images of these samples are shown in fig. S2]. Small size (in the submicron-scale) particles with an average lateral size of 0.6 μm (fig. S2A) were produced under low temperature (5°C) and high Cu-to-bpy ratio (1:5) conditions. The thickness of the particles increased almost linearly with the lateral size (Fig. 1E), indicating that the ratio of lateral size to thickness remained constant (~10) during particle formation, showing no variation with the Cu concentration, reaction temperature, and Cu-to-bpy ratio. This phenomenon can be attributed to the formation of equilibrium-shaped particles with a fixed lateral size-to-thickness ratio through a thermodynamically controlled synthesis under the reaction conditions used in this study.

**Fig. 2.**
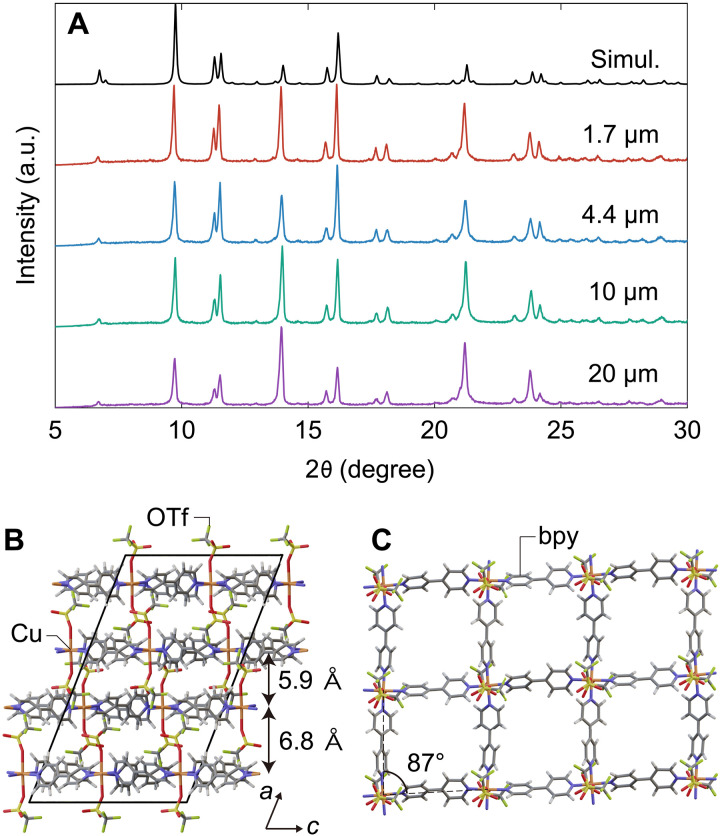
XRD pattern and atomic structure of the as-synthesized ELM-12. (**A**) XRD patterns of the as-synthesized ELM-12 particles with different lateral sizes. (**B**) Structure of the as-synthesized ELM-12, wherein the layer structures shown in (**C**) are stacked with alternating interlayer distances of 5.85 and 6.78 Å. The black line in (A) represents the theoretical XRD pattern of as-synthesized ELM-12, which is simulated on the basis of the structure shown in (B). a.u., arbitrary unit.

As-synthesized particles with different sizes synthesized by the microreactor-based process showed almost identical x-ray diffraction (XRD) patterns (Fig. 2A), confirming a size-independent framework structure. Moreover, these XRD patterns were similar to the XRD patterns of ELM-12 particles synthesized by the interfacial-reaction method after the adsorption of EtOH or water molecules (*36*). Thus, the microreactor-based direct-mixing procedure yielded ELM-12 particles that accommodated solvent molecules (EtOH and/or water). The black line in Fig. 2A and Fig. 2 (B and C) shows the theoretical XRD pattern and the structural model of ELM-12 in as-synthesized phase, respectively, which is essentially the same as the crystalline structure of ELM-12 reported by Kondo *et al.* [referred to as **2a** in (*14*)], but with minor modifications to the cell parameters (see Table 1) and subsequent relaxation of the atomic arrangement of the structural model through density functional theory (DFT) calculations. The as-synthesized structure comprises 2D layers with tetragonal grids, which are slightly distorted from squares (with an angle of ~87°; Fig. 2C), in a lattice arrangement stacked with alternating interlayer distances of 5.85 and 6.78 Å (Fig. 2B).

**Table 1.** Crystal data for ELM-12 in as-synthesized, α, β, and γ phases. α and β are guest-free structures, while γ is a N_2_-loaded structure.

	As synthesized	α	β	γ
*a* (Å)	27.115	25.635(3)	14.5454(7)	15.8072(17)
*b* (Å)	15.304	14.3299(6)	11.08362(31)	15.7972(6)
*c* (Å)	16.125	16.8858(22)	19.6618(11)	15.0322(15)
β (°)	111.309	110.147(5)	101.7544(26)	107.466(4)
Space group	*C*2/*c*	*C*2/*c*	*C*2/*c*	*Cc*
*Z*	8	8	4	4
Framework density (kg/m^3^)	1436.4	1537.6	1442.7	1250.4
*R*_wp_ (%)	–	5.64	6.89	7.68
∠Cu-Cu-Cu (°)	87.0	80.6	81.6	90.0
Layer spacing (Å)	5.85, 6.78	5.30, 6.73	6.38	7.17

Thus, the microreactor-based process enabled the size-controlled synthesis of ELM-12 particles with a reasonably narrow size distribution (with a lateral size within 0.6 to 23 μm and thickness within 0.06 to 2.3 μm) that can be used for particle size–dependent behavioral analyses.

### Particle size–dependent adsorption behavior

**Fig. 3.**
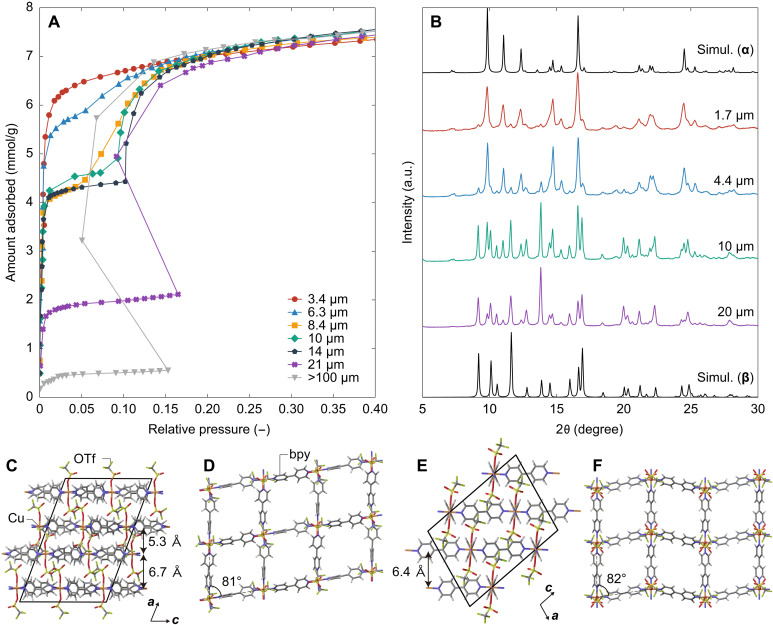
N_2_ adsorption isotherm, XRD pattern, and atomic structures of two guest-free phases of ELM-12. (**A**) N_2_ adsorption isotherms on ELM-12 at 77 K and (**B**) XRD patterns of ELM-12 with different lateral sizes under vacuum at 77 K. Guest-free structures of ELM-12 in (**C**) phase **α** and (**E**) phase **β**, wherein the layer structures shown in (**D**) and (**F**) are stacked.

**Fig. 4.**
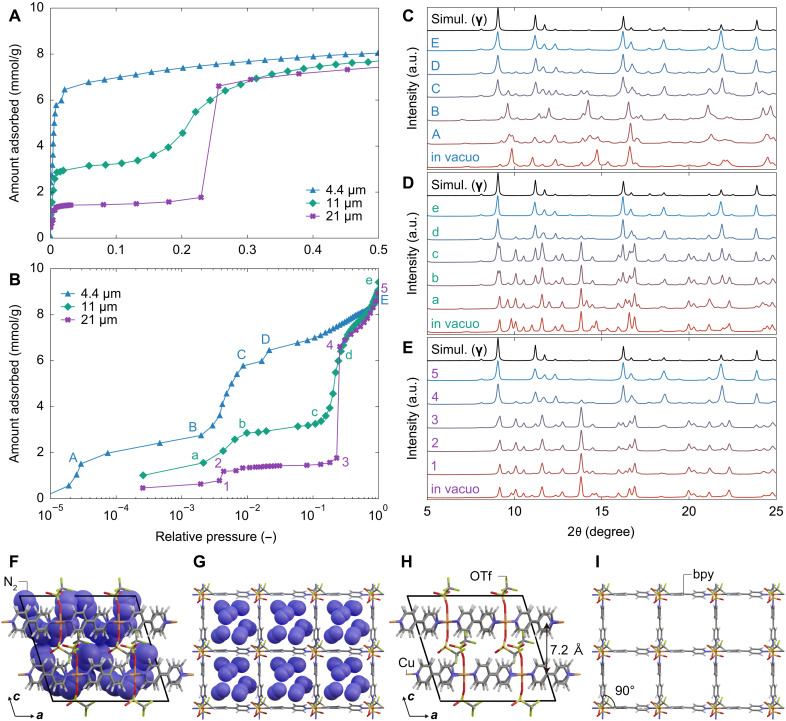
In situ XRD measurement and N_2_-loaded structure of ELM-12. N_2_ adsorption isotherms on ELM-12 particles with a lateral size of 4.4, 11, and 21 μm at 77 K in (**A**) linear and (**B**) logarithmic scales. In situ XRD patterns of ELM-12 particles with a lateral size of (**C**) 4.4, (**D**) 11, and (**E**) 21 μm recorded at the corresponding points indicated in (B). (**F**) Structure of ELM-12 in phase **γ**, wherein the layer structures shown in (**G**) are stacked with an interlayer distance of 7.17 Å. (**H**) and (**I**) represent N_2_-free visualizations of the structures shown in (F) and (G), respectively

Figure 3A shows the N_2_ adsorption isotherms of ELM-12 particles with different sizes at 77 K. Despite the identical structures of the as-synthesized ELM-12 particles (Fig. 2A), the adsorption isotherms varied remarkably with the particle size, exhibiting a considerable size dependence. ELM-12 particles with a size of 14 μm showed the typical double-step isotherm that is characteristic of flexible-robust–type systems in which the uptake in the first step is almost equal to that in the second step. Here, the ratio between the first and second steps changed with the particle size. The first step for 21-μm ELM-12 particles was almost half the first step for 14-μm particles, with an almost unchanged saturated adsorption amount. For the relatively large crystals (>100 μm) synthesized by the interfacial-reaction method (fig. S3), the first step was substantially smaller than the second step. In addition, the second step gradually changed on decreasing the particle size below 14 μm; eventually, 3.4-μm ELM-12 particles exhibited an apparently type I isotherm with an increased uptake at low pressures. The difference in the synthesis methods, the microreactor-based or the interfacial-reaction, did not have a major impact on the adsorption isotherms because particles, which were originally synthesized by the interfacial-reaction method and subsequently milled in a mortar, with sizes around tens of micrometers or smaller exhibited a similar adsorption isotherm to that of 21-μm particles synthesized by the microreactor-based process (fig. S4). Thus, a balance between robust and flexible characteristics can be controlled by tuning the particle size. Notably, stepped-back second-uptake adsorption isotherms were observed for large-sized ELM-12 particles (21 and >100 μm in size). This could be an apparatus artifact due to which a lower equilibrium pressure was measured after a large stepwise uptake owing to the small dead volume of the sample cell (~16 ml). No step back was measured on using an apparatus with a relatively large dead volume specific to the in situ XRD system (~85 ml), as later shown in Fig. 4A.

In situ XRD patterns of ELM-12 particles recorded at 77 K under vacuum after the activation procedure to remove solvent molecules are shown in Fig. 3B. The guest-free structures of ELM-12 varied with the particle size. A careful analysis of the in situ synchrotron XRD patterns indicated the existence of two guest-free structural phases, labeled structure **α** and **β**. The smaller sized (1.7 and 4.4 μm) particles mainly comprised **α**, while the larger sized (20 μm) particles predominantly comprised **β**. The XRD pattern of 10-μm ELM-12 particles indicated a mixture of both phases, and all the peaks in the pattern could be attributed to either of the two structures. Subsequently, initial structural models of **α** and **β** were constructed with reference to the guest-free structure of ELM-12 reported by Kondo *et al.* (*14*) and the open structure of ELM-11 (*37*), which is a structural analog of ELM-12 with BF_4_ anions as pillars in place of OTf anions (*38*), respectively, and optimized using the refinement method described in Materials and Methods. The structural models shown in Fig. 3 (C to F) indicate that **α** and **β** are structural isomers with stacking layer structures in which 2D grids are distorted into a rhombic shape at an angle of ~80° by the removal of solvent molecules. The interlayer distance and rhombic-grid alignment in the 2D layers were different in **α** and **β**; **α** showed alternate interlayer distances of 5.30 and 6.73 Å with a straight alignment of rhombic grids, while **β** showed a single interlayer distance of 6.38 Å with zigzag alignment. Straight and zigzag alignments are a common structural variation in tetragonal grids comprising Cu and bpy; the hydrated forms of ELM-11 comprise structural isomers with a straight and zigzag alignment of tetragonal grids (*39*). According to DFT calculations of potential energy, **β** is more stable than **α** by 34.7 kJ/mol, indicating that smaller ELM-12 particles are more likely to form the metastable structure **α** after the activation process.

The differences in the guest-free structures accounted for the variations in the adsorption isotherms, as demonstrated by in situ XRD measurements under different adsorption pressures (Fig. 4). Starting from **α** in vacuo, 4.4-μm ELM-12 transformed into an intermediate structure (labeled structure **α′**) through point A to B in the low relative pressure region of 10^−3^ (Fig. 4, B and C). New peaks appeared at point C after a steep increase in the adsorption amount, indicating a structural transition induced by N_2_ adsorption. Moreover, peaks corresponding to **α′** weakened at point C and lastly disappeared at point E, resulting in a N_2_-loaded structure. In contrast, for 21-μm ELM-12, the XRD patterns mainly comprising **β** remained almost unchanged until point 3 (*p*/*p*_0_ ~ 10^−1^) except for the peaks originated from **α**; after this point, **β** transformed into a N_2_-loaded structure accompanied with stepwise gas uptake from point 3 to 4 (Fig. 4, B and E). The N_2_-loaded structures of 21- and 4.4-μm ELM-12 were identical, indicating that the guest-free structures of both **α** and **β** eventually formed a large pore structure, labeled structure **γ**. For 11-μm ELM-12, **α** and **β** separately transformed into **γ** in such a way that the structural transition from **α** to **α′** to **γ** first proceeded through point a to b to c, followed by a transformation from **β** to **γ** through points c and d (Fig. 4, B and D). Therefore, the flexible-robust–type isotherms of ELM-12 can be attributed to the coexistence of two guest-free structures **α** and **β**. Type I–like uptake at low pressures corresponds to **α**, whereas the subsequent gate-type stepwise uptake corresponds to **β**.

The in situ synchrotron XRD patterns of 2.4-μm ELM-12 particles after the introduction of N_2_ gas were analyzed to characterize the N_2_-loaded structure **γ**. An initial structural model was constructed by referencing the layer structure of ELM-12 in the as-synthesized phase and optimized by refinement, resulting in the structural model shown in Fig. 4, F to I, and Table 1. The uptake of N_2_ molecules straightened the tetragonal grids back into square shape from the rhombic shapes of **α** and **β** and increased the interlayer spacing to 7.17 Å.

**Fig. 5.**
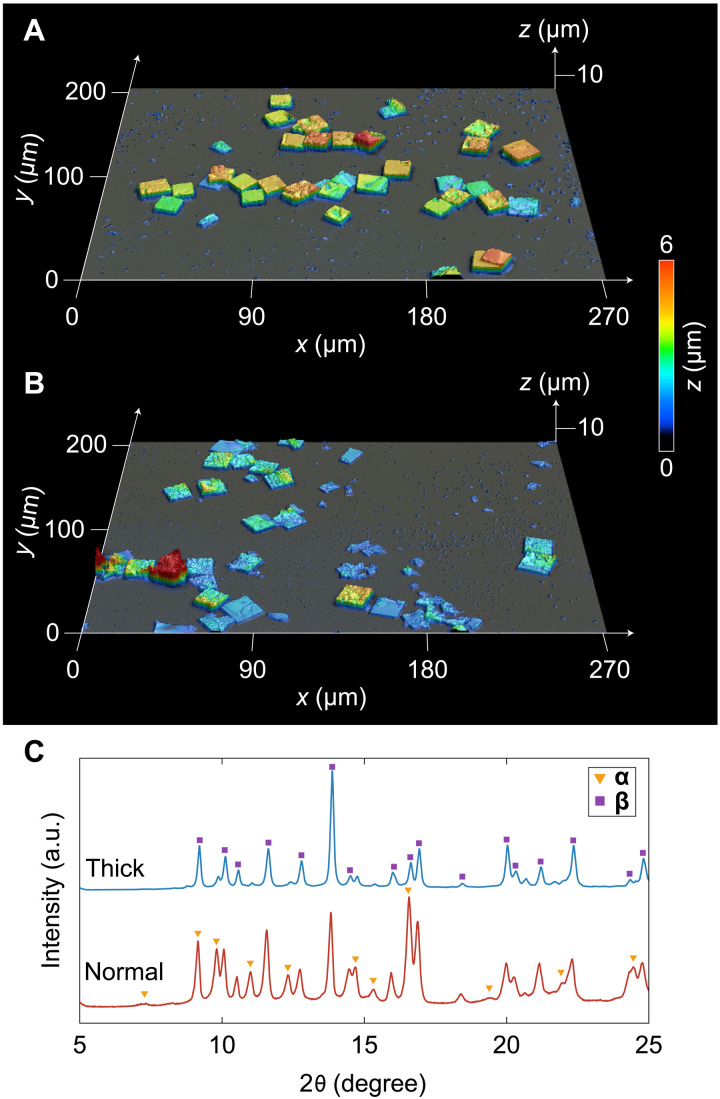
Controlling the thickness of ELM-12 particles while maintaining the lateral size. Laser scanning microscope images of ELM-12 synthesized (**A**) with pyridine for suppressed lateral growth and (**B**) under normal conditions. (**C**) XRD patterns of thick and normal ELM-12 particles with the same lateral size recorded under vacuum at 77 K after activation; triangles and squares represent peaks corresponding to **α** and **β**, respectively.

The ELM-12 particles showed a fixed lateral size-to-thickness ratio of 10 (Fig. 1E); consequently, lateral size reductions were accompanied with a concomitant reduction in thickness. To separately evaluate the effects of the lateral size and thickness on the adsorption behavior of ELM-12, relatively thick ELM-12 particles with a lateral size of 14 μm, thickness of 3.4 μm, and lateral size-to-thickness ratio of 4 were synthesized by adding pyridine as a modulator during ELM-12 synthesis (Fig. 5A). Pyridine suppressed the lateral growth of 2D grid sheets, facilitating growth in the thickness direction. Unlike the ELM-12 particles synthesized without pyridine with a lateral size of 14 μm and thickness of 1.5 μm (Fig. 5B), which contained two guest-free structures (**α** and **β**), the thicker ELM-12 particles mainly comprised the **β** phase in the guest-free state (Fig. 5C). Therefore, thickness influences the guest-free structure and corresponding adsorption behavior of ELM-12 to a greater extent than the lateral size. An analysis of the particle size dependence on the guest-free structure of ELM-12 in terms of thickness (Figs. 1E and 3) indicated that ELM-12 particles with a thickness of <0.5 μm (lateral size of 1.7 and 4.4 μm; Fig. 3B) predominantly formed **α**, and those with a thickness of >2 μm (lateral size of 20 μm; Fig. 3B) mainly comprised **β**, whereas those with an intermediate average thickness yielded a mixture of **α** and **β** (lateral size of 10 μm; Fig. 3B) owing to the intrinsic thickness distribution around the average particle thickness. As 14-μm ELM-12 exhibited a double-step isotherm with almost identical uptakes in the first and second steps (Fig. 3A), indicating equal amounts of **α** and **β**, an average thickness of 1.5 μm was identified as the critical thickness for the formation of the metastable guest-free structure **α** by the activation of as-synthesized ELM-12 particles.

### Guest-molecule dependence of guest-free structures

**Fig. 6.**
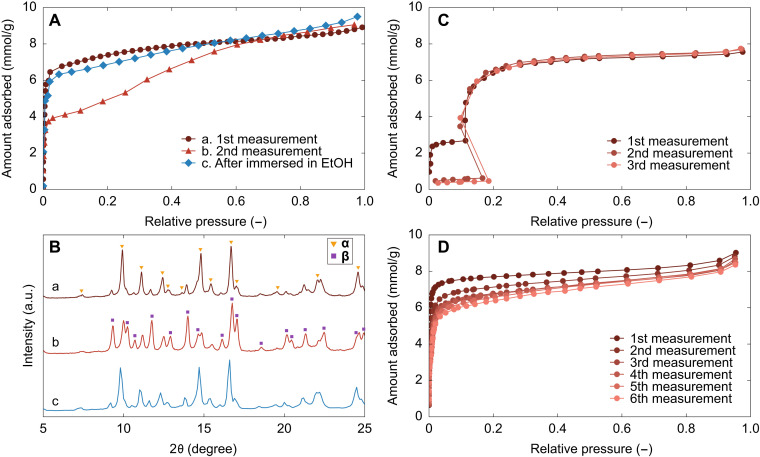
Guest-molecule dependence of guest-free structures of ELM-12. (**A**) N_2_ adsorption isotherms on 4.4-μm ELM-12 at 77 K under various conditions: (a) Data of the initial measurement using as-synthesized ELM-12, (b) data of the second measurement collected without air exposure, and (c) data of the third measurement after immersion in ethanol (EtOH). A pre-heat treatment was used before each measurement. (**B**) XRD patterns of ELM-12 under vacuum conditions before the adsorption measurements described in (A). Triangles and squares represent peaks corresponding to **α** and **β**, respectively. Repeated adsorption isotherms of N_2_ on (**C**) 19-μm and (**D**) 0.6-μm ELM-12 particles at 77 K.

An interesting feature of ELM-12 is the dependence of the guest-free structure on the type of guest molecules accommodated immediately before the activation procedure, which was confirmed by structural variation during repeated isotherm measurements (Fig. 6). The first measurement of 4.4-μm ELM-12 exhibited steep gas uptake at *p*/*p*_0_ ~ 10^−3^, resulting in an apparently type I isotherm, as the average thickness (0.4 μm) was lower than the critical thickness (1.5 μm) corresponding to the formation of the guest-free structure **α** [Fig. 6, A (a) and B (a)]. Although the second step was rather broad, the second measurement, which was recorded after the first isotherm measurement followed by activation without exposure to air, produced a double-step adsorption isotherm with gas uptake at a higher pressure of *p*/*p*_0_ ~ 10^−1^. This isotherm variation was attributed to the formation of the other guest-free structure **β** in the second measurement, as confirmed by in situ XRD measurements (Fig. 6B, b), indicating that ELM-12 particles with thicknesses below the critical thickness could form the **β** phase after N_2_ desorption. To confirm the recoverability of the guest-free structure, ELM-12 particles were immersed in ethanol after the second measurement; ethanol was used for immersion instead of water because ELM-12 particles partially dissolved in water. After the immersion, ELM-12 particles exhibited the type I–like adsorption isotherm induced by the formation of the **α** phase [Fig. 6, A (c) and B (c)]. Similarly, the adsorption isotherm of 19-μm ELM-12 with an average thickness of 2.2 μm, which was a double-step isotherm in the first measurement, showed an almost complete gate-type behavior in the second and third measurements with a suppression of the initial uptake at low pressures (Fig. 6C). No noticeable changes in the particle size and morphology were confirmed after the repeated adsorption measurements (fig. S5). Thus, ELM-12 particles within a certain thickness range formed the guest-free structures **α** and **β**, where the adsorption and desorption of nonpolar N_2_ molecules yielded **β**, while that of polar solvent molecules, such as ethanol and water, resulted in the formation of **α**. Thus, ELM-12 shows switchable flexible and robust adsorption characteristics that depend on the type of guest molecule accommodated immediately before the activation procedure. This structural variation upon guest removal is a unique behavior in that the guest-free structures **α** and **β** are both in closed/narrow pore phases, indicating the existence of two different structural transition paths from an open/large pore state. This is a contrast to the cases of some flexible MOFs showing the solvent-induced switchability in which the guest-free structure switches between closed/narrow pore and open/large pore phases along a single transition path (*40*, *41*), where the open guest-free structure is formed by keeping the open structure almost unchanged against the solvent removal. The formation of multiple closed guest-free structures for ELM-12 is possibly due to a high degree of structural freedom in the intra- and interlayer structure and the location of pillar molecules specific to the stacking layer structure.

The “guest-memory” switching of the guest-free structure was observed for ELM-12 particles that formed **α** after solvent molecule removal; accordingly, the upper limit of particle thickness for guest memory switching was identical as the critical thickness of 1.5 μm. The second adsorption isotherm of 4.4-μm ELM-12 (Fig. 6, A, b), in which the initial uptake at low pressures was almost half of the saturated adsorption amount, was used to investigate the lower limit of thickness for this phenomenon. Assuming that half of the 4.4-μm ELM-12 particles transformed into the **α** phase after the removal of N_2_ molecules, an average thickness of 0.4 μm was considered to be the lower limit for the guest-memory effect. Notably, the adsorption isotherms of submicron-sized ELM-12 particles (0.6 μm in lateral size and 0.06 μm in thickness) with a substantially lower thickness than 0.4 μm remained type I–like over six cycles with a gradual reduction in the initial increment on repetitive cycling to reach an almost identical saturated amount (Fig. 6D). Because no noticeable changes in the particle size and morphology were observed after the repeated adsorption measurements (fig. S5), the reduction in the initial increment was possibly due to the formation of the guest-free structure **β** in a small portion of submicron ELM-12 particles during the activation process, as the guest-free structure formation involved stochastic nature typified by energy barriers between the N_2_-loaded structure **γ** and the guest-free structures. Thus, N_2_ removal from ELM-12 particles slightly thinner than 1.5 μm induced the formation of the **β** phase, whereas N_2_ removal from substantially thinner ELM-12 particles (<0.4 μm) dominantly induced the formation of the **α** phase. Therefore, the guest-memory effect is observed in ELM-12 particles within a limited size range (4 to 14 μm) in the lateral dimension that corresponds to thicknesses within 0.4 to 1.5 μm.

### Mechanism of size-dependent flexible and robust adsorption characteristics

**Fig. 7.**
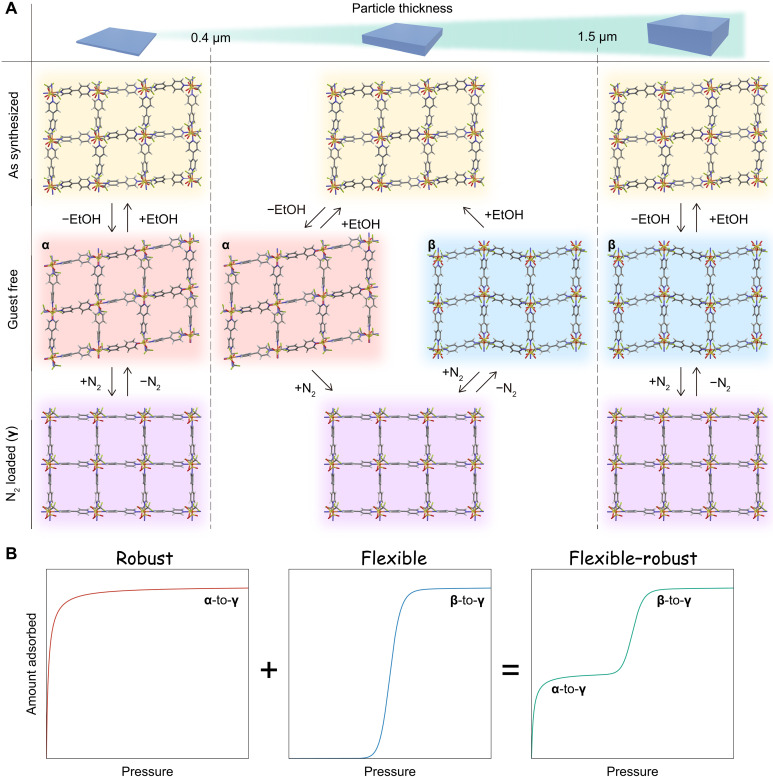
Size-dependent guest-memory switching of the flexible and robust adsorption characteristics of ELM-12. (**A**) Mechanism of size-dependent structural transformation. The key factor for controlling the guest-free phase is the particle thickness. Particles with a thickness of <0.4 μm are trapped in the metastable **α** phase, whereas those with a thickness of >1.5 μm transform into the stable **β** phase. Particles with intermediate thickness exhibit a guest-memory effect, in which solvent-encapsulated (as-synthesized) ELM-12 particles transform into the **α** phase, while N_2_-loaded ELM-12 (**γ**) particles transform into the **β** phase. (**B**) The flexible-robust nature of ELM-12 can be attributed to two gate openings: the **α**-to-**γ** transition at a low pressure (indicating the robustness of the system) and the **β**-to-**γ** transition at a moderate pressure (indicating the flexibility of the system).

The structural variations in ELM-12 particles of different sizes are summarized in Fig. 7A. Different sizes of the as-synthesized ELM-12 particles showed a similar crystalline structure, comprising tetragonal grids with interlayer distances of 5.85 and 6.78 Å (Fig. 2, B and C). In contrast, the guest-free structures of ELM-12 varied with the particle size (i.e., with the particle thickness). The activation procedure to remove solvent molecules (EtOH and/or water) from the as-synthesized structures and subsequent cooling to 77 K transformed the particles thinner than the critical thickness of 1.5 μm into the metastable guest-free structure **α** with linearly aligned rhombic grids (Fig. 3, C and D), whereas thick particles (>1.5 μm in thickness) transformed into the stable structure **β** with zigzag grid alignment (Fig. 3, E and F). Upon N_2_ adsorption at a relative pressure of ~10^−3^, **α** transformed into the N_2_-loaded structure **γ** (Fig. 4, F to I), whereas the **β**-to-**γ** transition occurred at a higher relative pressure of 10^−1^. As the adsorption branches in gate-adsorption processes are kinetically controlled (not thermodynamically) (*42*–*44*), a higher pressure for the **β**-to-**γ** transition than the **α**-to-**γ** transition indicates a higher energy barrier between the structures **β** and **γ**. Detailed analysis indicated that the structural transformation from **β** to **γ** involved an exchange of the relative positions of the OTf anions (see fig. S6); this exchange is an energetically expensive process that occurs through an unstable transition state. As the transformation of **α** to **γ** did not involve this exchange, the high energy barrier of the transformation was attributed to the structural dissimilarity between **β** and **γ** in terms of the relative positions of OTf anions.

The formation of **α** is favored in small-sized ELM-12 despite the higher stability of **β** than **α** in the bulk phase. Two possible scenarios can be considered for **α** formation to be preferred over that of **β**. The first scenario is based on the surface effect accompanied by particle downsizing. Owing to a higher framework density (Table 1), **α** offers a smaller surface-to-volume ratio than **β**. Hence, for small-size ELM-12 particles in which the surface effect remarkably influences the system stability, the formation of **α** can be preferred over that of **β** despite the higher stability of the latter in the bulk phase. However, simple calculations indicate that the density difference is insufficient to ensure system stabilization, making this scenario improbable (see Supplementary Text). The second scenario, which is based on the kinetic effect, is the most probable explanation for the favored formation of **α**. According to this scenario, small-sized ELM-12 particles are trapped in the metastable state upon guest removal. Because the structural transformation is assumed to proceed during the guest removal process, smaller particles inevitably have a shorter time for the transformation because of a smaller number of guest molecules per particle. Because the as-synthesized structure of ELM-12 is more similar to **α** than **β** in terms of the alternation of the interlayer distance, straight alignment of the tetragonal grids, and the relative positions of OTf anions, the transformation of the as-synthesized state into **α** is expected to involve a lower energy barrier than transformation into **β**; accordingly, smaller particles are more easily trapped in the metastable **α** structure.

Notably, the critical thickness for **α** formation varies with the type of guest molecule; the critical thickness for N_2_ guest molecules (0.4 μm) is lower than that for solvent molecules (1.5 μm), possibly owing to the degree of structural similarity between the guest-loaded structures and **α**. That is, the as-synthesized structure including solvent molecules is more similar to **α** compared with a similarity between **γ** (which accommodates N_2_ molecules) and **α**, resulting in a lower energy barrier for the transformation into **α** from the as-synthesized structure than the **γ**-to-**α** transformation. The results of the study indicate that the critical thickness is a vital parameter influencing the guest-memory switching effect; guest-free structures within a certain thickness range undergo a transformation that depends on the history of the guest-molecule type immediately before the activation process. For ELM-12 particles with thicknesses within 0.4 to 1.5 μm, the removal of solvent molecules from the as-synthesized structure yields **α**, whereas the removal of N_2_ molecules from **γ** forms **β**. Hence, the **α**-to-**γ** transition is not reversible for particles in this thickness range, whereas it is reversible in substantially thinner particles (<0.4 μm).

The structural variation of ELM-12 particles directly influenced their adsorption behavior. The structural transition of **α** resulted in a rapid increase in N_2_ uptake at a very low-pressure region, which apparently appeared as a type I isotherm; consequently, the robust nature of ELM-12 is attributed to the gate adsorption accompanying the **α**-to-**γ** transition (Fig. 7B). The flexible nature of ELM-12 corresponded to the gate adsorption caused by the **β**-to-**γ** transition at a relative pressure of 10^−1^ (Fig. 7B). The amounts of particles in the **α** and **β** phases after the activation process determined the magnitudes of the first and second steps in the resultant isotherms, respectively. Double-step isotherms with equal uptakes for the first and second steps, which are typical of flexible-robust–type systems, appear for particles with lateral sizes of ~15 μm as the average thickness is near the critical thickness of 1.5 μm, while ELM-12 particles that are thinner and thicker than the critical thickness yield type I-like (characteristic of robust systems) and stepwise (characteristic of flexible systems) isotherms, respectively. Furthermore, through guest-memory switching, ELM-12 particles that originally exhibited flexible-robust isotherms showed the characteristics of a flexible system through the introduction and subsequent removal of N_2_ molecules. As the critical thickness varies with the type of guest molecule, the thickness range for guest-memory switching can be further optimized. Therefore, the precise control of the particle size and the suitable selection of guest molecules for the guest-memory effect enable the fine regulation of the gate-adsorption characteristics of ELM-12 from robust type to flexible robust type to flexible type.

**Fig. 8.**
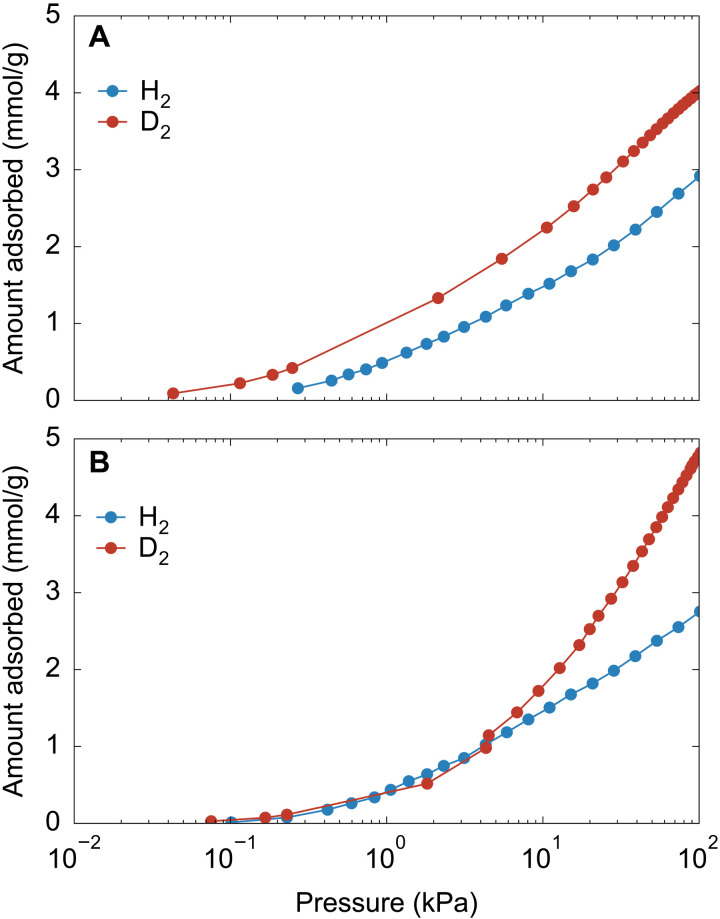
Potential application of ELM-12 enhanced by particle size control. (**A**) H_2_ and D_2_ adsorption isotherms at 77 K in particles with a lateral size of 0.6 μm, wherein ELM-12 adsorbs D_2_ at lower pressures than H_2_. (**B**) H_2_ and D_2_ adsorption isotherms at 77 K in particles with a lateral size of 19 μm, where the D_2_ adsorption isotherm exhibits the gate-opening behavior at 4 kPa.

As described above, the guest-free structure **α** is not a rigid framework; instead, it is a flexible system that undergoes structural transition at a very low pressure. During the transformation of **α**, the small amount of stabilization provided by guest-molecule adsorption compensated for the stability difference between the guest-free and open states, indicating that **α** shows a high–molecular recognition ability through pore structure opening in response to weak interactions. Therefore, the flexible nature of **α** can be used for the separation of molecules with similar properties. Model systems comprising H_2_ and D_2_ were used to investigate the feasibility of the concept; the adsorption isotherms of H_2_ and D_2_ on submicron (0.6-μm) and micron (19-μm) ELM-12 particles were recorded at 77 K (Fig. 8). Note that 19-μm ELM-12 particles were pretreated with N_2_ adsorption and desorption to increase the **β** phase by taking advantage of the guest-memory switching characteristics (Fig. 6C). ELM-12 (0.6 μm) whose guest-free structure predominantly comprised the **α** phase showed larger uptakes of D_2_ than those of H_2_ over the entire pressure range, whereas 19-μm ELM-12 with the **β** phase as the guest-free structure exhibited a stepwise increase in D_2_ uptake at 4 kPa after which the amount of adsorbed D_2_ increased more rapidly than those of H_2_, indicating a gate-opening behavior selectively responding to D_2_. Considering that adsorption profiles of 19-μm ELM-12 were almost identical between H_2_ and D_2_ at pressures below the gate opening at 4 kPa (Fig. 8B), the larger uptakes of D_2_ on 0.6-μm ELM-12 is attributable to a gate opening of **α** in a very-low-pressure region. ELM-12 was thus demonstrated to exhibit an isotope-selective structural responsivity (*45*–*47*), and the sensitivity is higher for smaller submicron-sized ELM-12 particles. Therefore, these results demonstrate high potential of ELM-12 for separations involving light gases with weak interactions and highlight the importance of the size-controlled synthesis around the critical size for enhanced separation performance.

## DISCUSSION

This study investigated the effects of particle size on the flexible-robust adsorption characteristics of ELM-12. ELM-12 particles of different sizes with a lateral dimension of 0.6 to 23 μm and a thickness of 0.06 to 2.3 μm having a fixed ratio of lateral size to thickness as ~10 were synthesized using a microreactor-based process. Efficient mixing by this process enabled the formation of ELM-12 particles with narrow size distributions. Although the as-synthesized structures were identical, regardless of particle size, their adsorption and structural-transition behavior showed remarkable particle size dependence. In situ XRD measurements revealed that type I–like uptake at low pressures in a double-step isotherm, corresponding to the robust nature of the system, was caused by gate-type adsorption from the metastable guest-free structure **α** to the N_2_-loaded structure **γ** at *p*/*p*_0_ ~ 10^−3^. The second step, corresponding to the flexible nature of the system, was due to the structural transition of the stable guest-free structure **β** to **γ** at *p*/*p*_0_ ~ 10^−1^. The coexistence of the two flexible guest-free structures **α** and **β** resulted in a double-step isotherm; the amount of each structure determined the uptake at each step. The structure **α** was observed in small-sized particles, while **β** was observed in large-sized particles in which the particle thickness (not the lateral size) predominantly influenced the guest-free structure. The critical thickness for the formation of the metastable phase **α** varied with the guest molecule to be removed, with a value of 1.5 μm for solvent molecules and 0.4 μm for N_2_. The difference in critical thickness emerged as a vital parameter influencing the guest-memory effect; ELM-12 particles within a specific thickness range exhibited different guest-free structures and adsorption behaviors depending on the guest-molecule history. This study highlights the importance of the size-controlled synthesis of flexible-robust MOFs in fine-tuning the adsorption characteristics of these systems for targeted separation applications. The high–molecular recognition ability of flexible-robust MOFs is attributed to their guest-free structure, which is suitable for specific hydrocarbons, along with dynamic structural adjustment (*13*, *18*). Therefore, controlling the structural flexibility of MOFs through particle size tuning can enhance the separation efficiency of such systems, expediting their industrial application.

Notably, this study does not completely elucidate the mechanism underlying the guest-memory switching effect. However, the results of this study indicate that ELM-12 particles within a specific size range can form two different guest-free structures, indicating that the kinetics of these transformations are possibly related and that the height of the energy barriers between the structures before and after the transitions could be a key factor in determining the final guest-free structure, as previously discussed in a flexible MOF system (*41*). Hence, future studies should investigate these structural transition paths. In addition, the temperature dependence of structural stability could be another key contributor to the guest-memory effect because the activation process to remove guest molecules involves a temperature variation. This indicates that the rate of temperature variation could be an operational parameter for controlling the guest-memory effect because guest-free structure formation is kinetically controlled. Relevant research is currently ongoing and will be reported elsewhere.

## MATERIALS AND METHODS

### Chemicals

Cu(OTf)_2_ (98%) and pyridine (99.8%) were purchased from Sigma-Aldrich Chemical Co., ethanol (EtOH, 99.5%) was purchased from Kishida Chemical Co., and bpy (98%) was purchased from Tokyo Chemical Industry Co. Ltd. All chemicals were used without further purification. Aqueous solutions were prepared in ultrapure water (resistivity > 18 megaohm·cm) from a water purifier system (Sartorius, Germany).

### Synthesis of ELM-12 particles

An aqueous solution of Cu(OTf)_2_ and an EtOH solution of bpy were pumped into a central collision-type microreactor using a two-channel syringe pump at a flow rate of 20 ml/min through each syringe. The central collision-type microreactor comprises submillimeter sized channels (~100 μm) and demonstrates an excellent mixing performance with a mixing time of 0.3 ms, which is 100 times shorter than that of batch-type mixing (*30*). The volume of each solution was typically 10 ml. The two solutions underwent rapid mixing in the microreactor, forming ELM-12 particles via a coordination reaction. The mixed solution was collected in a vial through a tube connected to the microreactor outlet and maintained under quiescent conditions for 15 min. The reaction was assumed to be complete within 15 min because the resultant particle size did not vary on increasing the reaction time further (fig. S7). The synthesized particles were collected by vacuum filtration using a polycarbonate membrane filter with a pore size of 0.8 μm, washed with 1 to 3 ml of EtOH, and dried under vacuum overnight. The Cu concentration and Cu-to-bpy ratio were varied within 25 to 40 mM and 1:2 to 1:5, respectively, while the reaction temperature was varied within 5° to 30°C by immersing the microreactor and vial into a water bath. ELM-12 particles were formed in yields of 50 to 60% (on a Cu basis). A semi-batch–type experiment in which an aqueous solution of Cu(OTf)_2_ (10 ml) was poured into an EtOH solution of bpy (10 ml) in a vial at a flow rate of 50 ml/min followed by stirring at 1500 rpm for 15 min was used to analyze the effect of the mixing procedure on the synthesis. The ELM-12 yield of the semi-batch synthesis (40 to 45%) was slightly lower than that of the microreactor process. Relatively large (>100 μm) ELM-12 crystals were formed on using the interfacial synthesis strategy reported by Kondo *et al.* (*14*) (fig. S3).

In separate experiments, pyridine was added as a modulator to regulate particle growth. An aqueous solution of Cu(OTf)_2_ (30 mM) was mixed with a premixed solution of bpy (60 mM) and pyridine (20 mM) in EtOH in the microreactor at a flow rate of 20 ml/min through each syringe at 20°C. The mixed solution was collected in a vial and kept stationary for 15 min, followed by the vacuum filtration, EtOH washing, and vacuum drying overnight.

### Characterization

SEM images were recorded using a JSM-6700F field-emission scanning electron microscope (JEOL Ltd. Japan). The synthesized ELM-12 particles were coated with Au (thickness: 7.0 nm) using a Q150TS turbo-pumped sputter coater (Quorum Technologies Ltd. UK) before imaging to minimize charging problems. The average lateral size and standard deviation (SD) of the resultant ELM-12 particles were calculated by measuring the size of at least 100 particles in the SEM images. The particle thickness was measured using a confocal laser scanning microscope VK-X100 (Keyence Corp. Japan) to estimate the average and SD of 20 particles. To estimate the thickness of submicron-sized ELM-12 particles, an atomic force microscopy instrument (MFP-3D Origin, Oxford Instruments, UK) was used to record height images of ELM-12 particles deposited on a mica substrate by scanning in air. The N_2_, H_2_, and D_2_ adsorption isotherms on ELM-12 at 77 K were measured using a BELSORP-MINI (MicrotracBEL Corp., Japan) automated adsorption apparatus. Before each isotherm measurement, the ELM-12 particles were activated at 363 K for 90 min under vacuum (<10 Pa). Powder XRD patterns were recorded using an x-ray diffractometer (UltimaIV/285/DX, Rigaku Corp. Japan) with Cu Kα radiation (40 kV and 40 mA) at a scan rate of 5.0°/min and step size of 0.05°. The in situ XRD patterns of ELM-12 particles during the N_2_ adsorption were measured using BELSORP-18 (MicrotracBEL Corp.) and an x-ray diffractometer (SmartLab; Rigaku Corp.) with a cryostat equipped with a single-stage Gifford-McMahon refrigerator with Cu Kα radiation (45 kV and 200 mA) at a scan rate of 2.5°/min and step size of 0.01° or on the BL02B2 beamline of the SPring-8 synchrotron facility, Japan, using incident x-rays with a wavelength of 0.1000388 nm and a Debye-Scherrer–type multi-MYTHEN detector system (*48*).

### Structural analysis

The initial structures of ELM-12 in the guest-free and N_2_-loaded phases were constructed by analyzing the in situ synchrotron XRD patterns of ELM-12 using the indexing program Conograph (*49*), referencing the frameworks of the ELM series (as described in Results). Subsequently, these structures were refined by a method combining Rietveld analysis with potential energy calculations, generating crystallographically and thermodynamically feasible structures (*37*, *50*). The refinement method used in this study involves structural optimization to minimize the evaluation function *E* (= *wR*_wp_ + *U*), where *R*_wp_ is the reliability factor that indicates the agreement between the observed and calculated XRD patterns, *U* is the potential energy, and *w* is a weighting parameter. GSAS-II (*51*) and CP2K (*52*) were used to evaluate *R*_wp_ and *U*, respectively; the detailed method and results are provided in Supplementary Text.

### DFT calculations

The relative stability of the two guest-free structures of ELM-12 was evaluated using DFT including dispersion correction (DFT-D) calculations with the CP2K software (*52*). A cutoff energy of 400 rydberg was used along with the Perdew–Burke–Ernzerhof (PBE) functional and DZVP-MOLOPT-SR basis set. In addition, the D3(BJ) method proposed by Grimme *et al.* (*53*) was applied. The convergence criterion for energy calculations was set to be less than 10^−6^ hartree. The structures determined in the “Structural analysis” section were optimized while maintaining the space group symmetry with the aid of Atomic Simulation Environment (*54*) Python libraries, and optimization was continued until the maximum force exerted on the atoms was less than 0.005 eV/Å. Γ-point sampling in the Brillouin zone with a conventional cell, as listed in Table 1, was used during geometrical optimization. In addition, Γ-point sampling with a (1 × 2 × 2) supercell for **α** and (2 × 2 × 2) supercell for **β**, where **α** and **β** represent the guest-free structures of ELM-12, was used for relative stability calculations, thereby improving the optimization accuracy.
