# Supplementary material for: Size-dependent guest-memory switching of the flexible and robust adsorption characteristics of layered metal-organic frameworks
Source: Sci Adv. 2024 Dec 6;10(49):eadr1387. doi: 10.1126/sciadv.adr1387 (PMC11623303; doi:10.1126/sciadv.adr1387)
Supplement: Supplementary file 1 — Supplementary Text Figs. S1 to S11 [file sciadv.adr1387_sm.pdf]

Science Advances
AAAS

Supplementary Materials for

Size-dependent guest-memory switching of the flexible and robust adsorption characteristics of layered metal-organic frameworks

Satoshi Watanabe *et al.*

Corresponding author: Satoshi Watanabe, nabe@cheme.kyoto-u.ac.jp;
Shotaro Hiraide, hiraide@cheme.kyoto-u.ac.jp

Sci. Adv. **10**, eadr1387 (2024)
DOI: 10.1126/sciadv.adr1387

This PDF file includes:

Supplementary Text
Figs. S1 to S11

Supplementary Text

Structural analysis

Our structural refinement method includes the following steps (*33, 44*):

1 Preparation

First, we determine cell parameters using the indexing program Conograph (*43*) and refine cell parameters and parameters for the peak profile function with Pawley method using GSAS-II (*45*). Then, an initial structure model is constructed along the determined cell parameters with referencing structures of ELM series and optimized through standard DFT-D calculations using CP2K (*46*).

2 Monte Carlo (MC) simulation

As the first step of the refinement process, MC simulations are employed to allow large movements to escape from local minima possibly existing in the evaluation function E (= wR_{wp} + U, where R_{wp} is the reliability factor indicating the agreement between the observed and calculated XRD patterns, U is the potential energy, and w is a weighting parameter). Here, molecules such as 4,4'-bipyridine and OTf are treated as rigid bodies. Their positions and rotational angles are updated according to probability P,

$$P = \min\left[1, \exp\left(-\frac{\Delta E}{RT}\right)\right]$$

where ΔE is the change in the evaluation function before and after the trials, R is the gas constant, and T is the temperature. The evaluation of R_{wp} uses the Python scripting interface of GSAS-II without any internal parameter updates by the GSAS-II's routines. That is, this step is responsible for managing the structure variables $\boldsymbol{p}$ (molecular positions/angles and the scaling parameter s for XRD pattern intensity) while keeping other parameters (peak profiles, background, and cell size) constant. Evaluations of U follow using CP2K under the conditions defined in Methods, except for the convergence criterion for SCF, which is set at 10^{-4} hartree. Note that trial moves for molecules are limited to satisfy the space group symmetry of the structure, whose detailed explanation can be found in our previous studies (*33, 44*).

3 Molecule level minimization

This stage optimizes E by adjusting the structure variables $\boldsymbol{p}$, utilizing the L-BFGS method. This step directly follows the last MC simulation configuration to further refine the structure.

4 Atomic level minimization

Apart from the rigid body approach of earlier steps, this step adjusts all atoms' positions within the structural model to minimize E again by applying the L-BFGS method. This approach allows for finer structural refinements. In this step, the convergence criterion for SCF in DFT calculations is set at 10^{-6} hartree.

5 Product Rietveld analysis

Finally, Rietveld analysis is performed using GSAS-II for the last configuration of step 4 , where all parameters except for the atomic positions are optimized to evaluate the final R_{wp}.

The weighting factor, w, balances the XRD pattern conformity and potential energy within the evaluation function E. In the present study, we determined w = 50 kJ/mol-Cu in a trial-and-error manner, which means that a 1% increase in R_{wp} corresponds to an energy increase of 50 kJ/mol-Cu. In addition, although the potential energy derived from CP2K is the value per simulation box, we converted it into the value per Cu ion (i.e., kJ/mol-Cu) to equalize the impact of wR_{wp} in different sizes of simulation boxes, given that the conventional cell of **α** has 8 Cu ions while those of **β** and **γ** have 4 Cu ions, where **α** and **β** are guest-free structures and **γ** is a N_2-loaded structure. In the initial MC simulation, T was set at 1000 K and reduced by 100 K every

100 steps until reaching 100 K through 1000 optimization steps. Subsequent minimization persisted until either the absolute maximal change in the evaluation function per p_i ($\partial E/\partial p_i$) falls below 10^{-2} kJ/mol, or 1000 steps were exceeded. Final optimizations continued until achieving an "effective" force including the R_{wp} factor on any atom below 0.005 eV/Å. In the structural analysis for **γ**, the Preparation step was conducted only for the framework structure, followed by the grand canonical MC simulation to determine the initial configurations of adsorbed N_2 molecules (see Ref. 44 for more calculation details).

We utilized in situ XRD patterns of 2.4 μm ELM-12 in vacuo, 21 μm ELM-12 in vacuo, and 2.4 μm ELM-12 at 100 kPa-N_2 to analyze **α**, **β**, and **γ**, respectively. For the analysis of guest-free structures, considering the guest memory effect, the XRD pattern of the 2.4 μm ELM-12 was measured immediately after activation from the as-synthesized phase to enrich the **α** phase. Similarly, the XRD pattern of the 21 μm ELM-12 was measured immediately after activation from the N_2-loaded phase to enrich the **β** phase. These patterns were measured at 77 K. Since the XRD pattern of 2.4 μm ELM-12 in vacuo exhibits peaks corresponding to both **α** and **β**, we initially analyzed the structure of **β** using that of 21 μm ELM-12 in vacuo. Subsequently, the structure of α was optimized through multiphase analysis, maintaining **β** as fixed.
Fig. S8 shows the developments of ΔE (= $E - E_0$), R_{wp}, and ΔU during our structural refinement process for **γ**. The R_{wp} value decreased with U remaining almost unchanged, indicating that the structure was crystallographically optimized while maintaining thermodynamic validity. The results of final Rietveld analysis for **α**, **β**, and **γ** are shown in Figs. S9–S11, respectively.

Stability estimation of a particle with α and β structures

Let us assume a particle, which forms the structure **α**, with a side length L [m] and thickness h [m]. The bulk free energy of the particle is expressed as $L^2hg_{\text{bulk-}\alpha}$, where $g_{\text{bulk-}\alpha}$ [J/m^3] is the bulk free energy of **α**, and the surface free energy of the particle is $2L^2g_{\text{layer}} + 4Lhg_{\text{side}}$, where g_{layer} [J/m^2] and g_{side} [J/m^2] are the surface free energies of the layer surface and the side surface, respectively. The total free energy of the particle, G_α, is given by the sum of the bulk and surface free energies.

$$G_\alpha = L^2hg_{\text{bulk}-\alpha} + 2L^2g_{\text{layer}} + 4Lhg_{\text{side}}$$

For a particle with the structure **β**, the total free energy G_β is similarly given by the following equation under an assumption that the surface free energies are the same as those of **α**.

$$G_\beta = L^2h'g_{\text{bulk}-\beta} + 2L^2g_{\text{layer}} + 4Lh'g_{\text{side}}$$

where h' is the thickness and $h' = 1.06h$ based on the structural properties (Table 1 in the manuscript). For the **α** phase to be more stable than **β**, $G_\alpha < G_\beta$ must be satisfied, resulting in the following inequation with the geometrical relationship $L = 10h$ of ELM-12.

$$h < \frac{g_{\text{side}}}{41.7\left(g_{\text{bulk}-\alpha} - 1.06g_{\text{bulk}-\beta}\right)}$$

Although $g_{\text{bulk-}\alpha}$ and $g_{\text{bulk-}\beta}$ are not available, the difference between the bulk free energies of **α** and **β** is estimated to be on the order of 10^4 J/mol-Cu based on the potential energies calculated by DFT-D calculations. Assuming the number density of Cu in the framework to be 1 /nm^3, the bulk free energy difference, $g_{\text{bulk-}\alpha} - g_{\text{bulk-}\beta}$, is roughly estimated as 2×10^7 J/m^3. This leads to the following.

$$h < \frac{g_{\text{side}}}{41.7\left(g_{\text{bulk}-\alpha} - 1.06g_{\text{bulk}-\beta}\right)} < \frac{g_{\text{side}}}{41.7\left(g_{\text{bulk}-\alpha} - g_{\text{bulk}-\beta}\right)} = \frac{g_{\text{side}}}{41.7 \times (2 \times 10^7)}$$

According to the inequation, the thickness h is calculated to be smaller than 1×10^{-11} m if 10^{-2} J/m^2 is taken for g_{side}, which is a typical value for the surface free energy of organic materials. Therefore it is unlikely that the downsizing of particles induces the stability reversal between the structures α and β, and accordingly the structure β is assumed to be more stable than α over an entire thickness range.

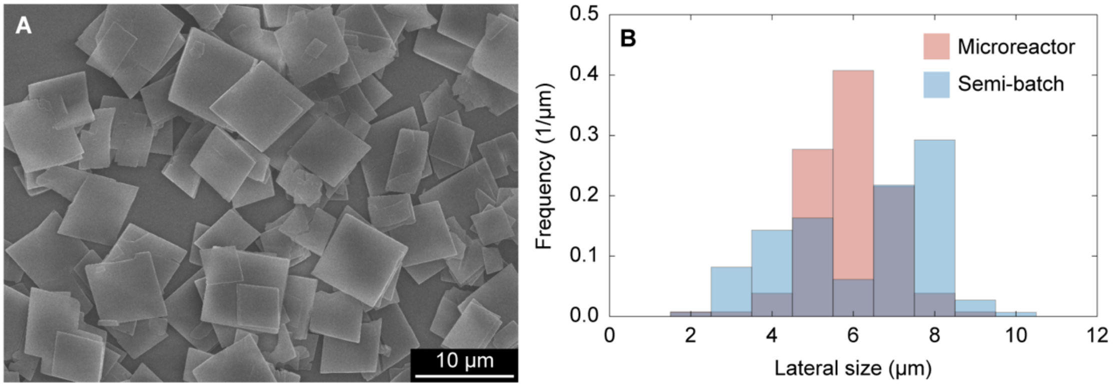

Fig. S1.

Synthesis results for the ELM-12 particles using semi-batch-type mixing. (**A**) A representative SEM image. (**B**) Lateral-size distribution of ELM-12 particles.

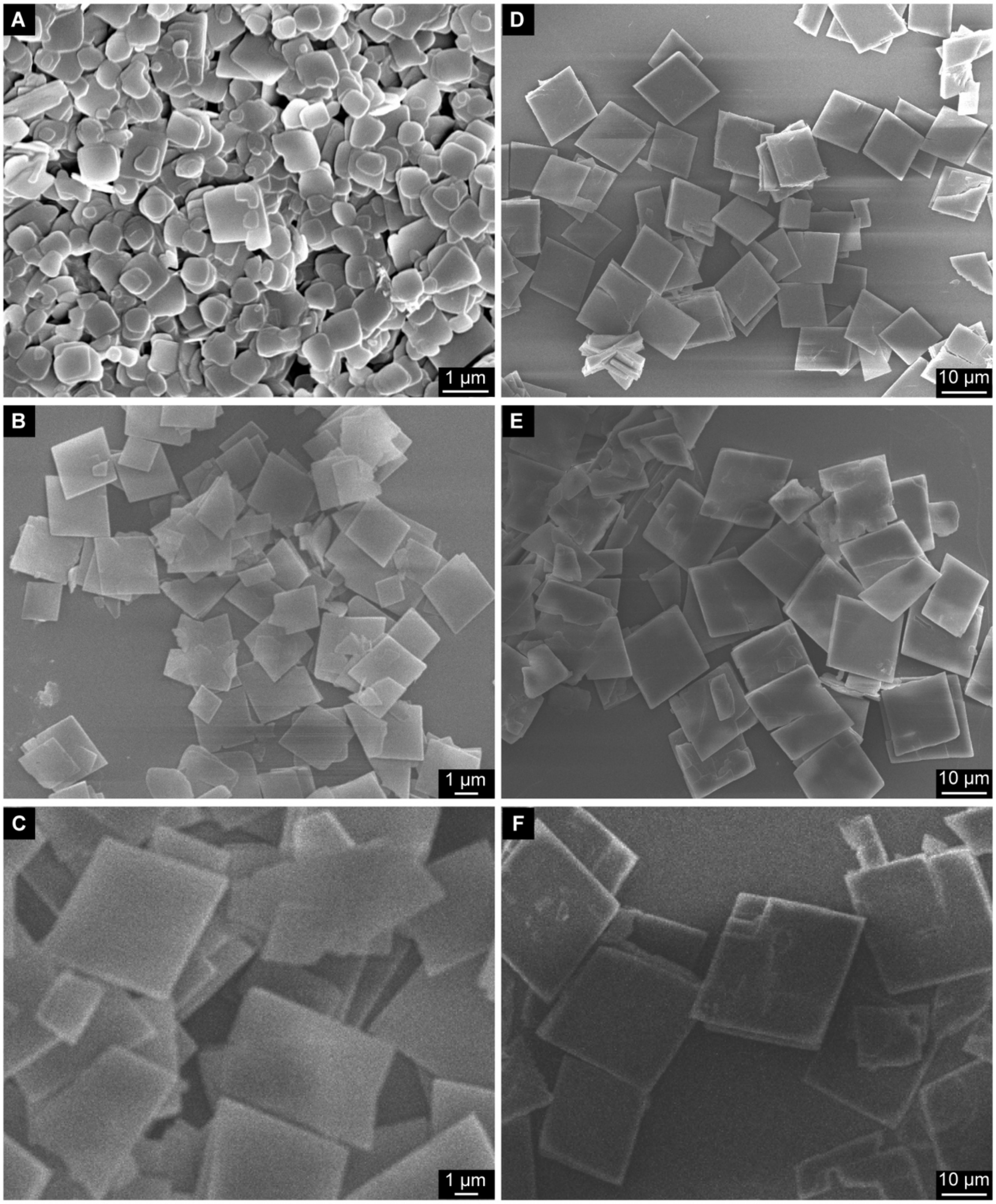

Fig. S2.

SEM images of ELM-12 particles synthesized via a microreactor. The average lateral sizes are as follows: (**A**) 0.6 µm, (**B**) 2.3 µm, (**C**) 6.0 µm, (**D**) 12 µm, (**E**) 16 µm, and (**F**) 23 µm.

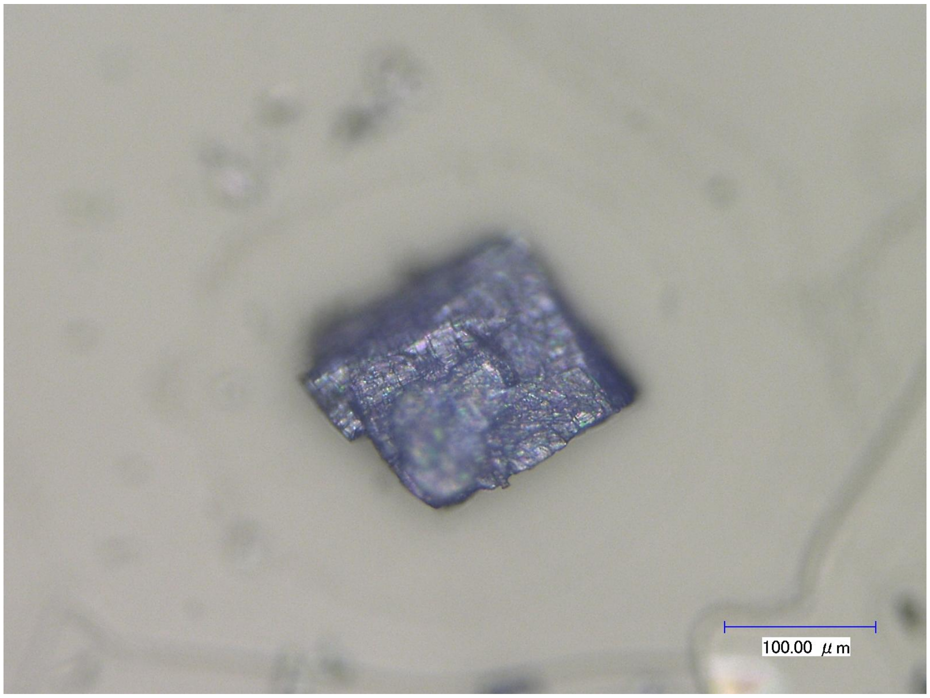

Fig. S3.
Optical image of an ELM-12 crystal synthesized via the interfacial-reaction method.

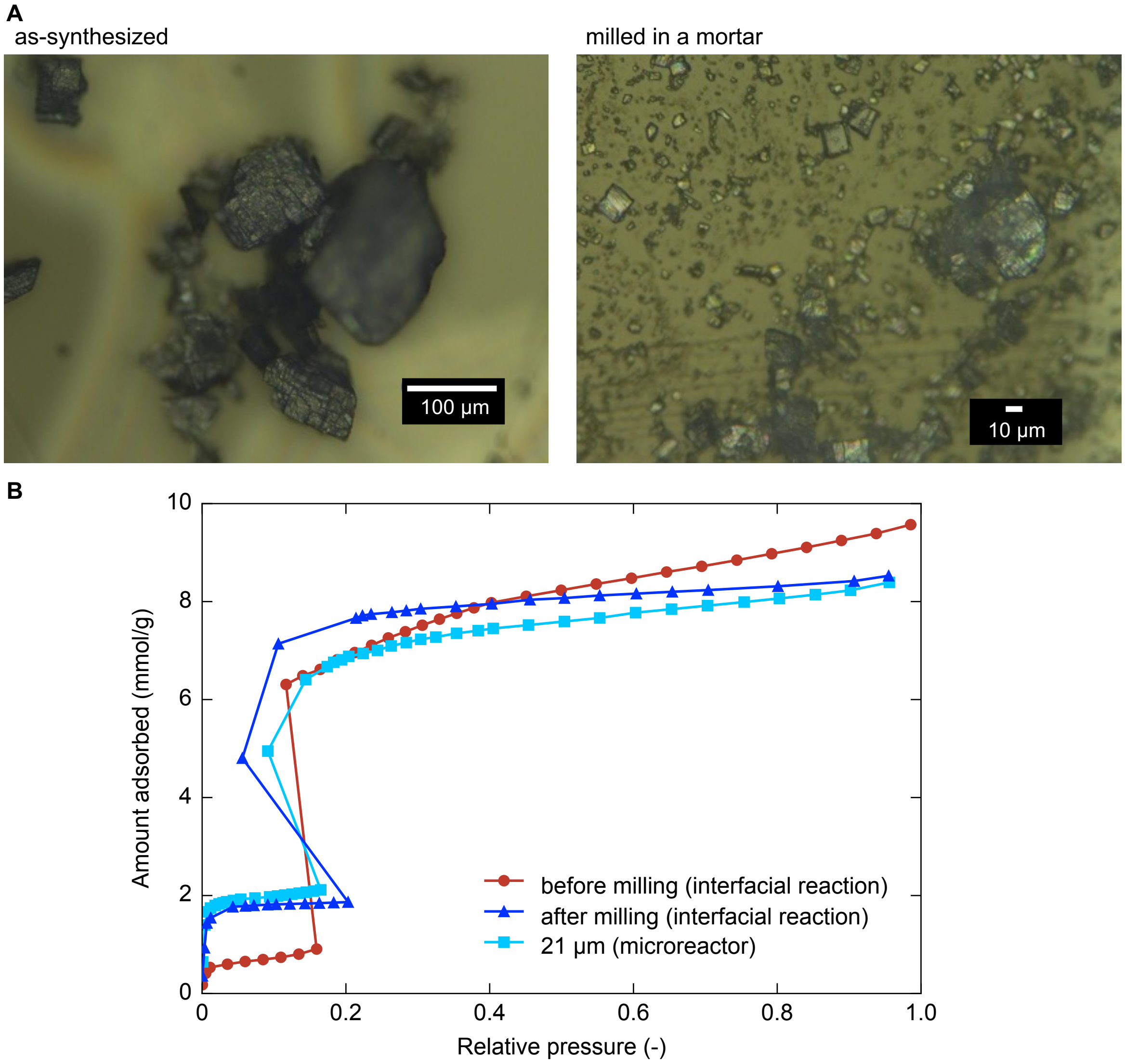

Fig. S4.
Effect of downsizing of large ELM-12 crystals synthesized by the interfacial-reaction method. (**A**) Optical microscope images of particles before (as-synthesized) and after being milled in a mortar. (**B**) N_2 adsorption isotherms at 77 K on the large crystals and particles milled in a mortar along with that of 21-µm particles synthesized by microreactor-based process. As the adsorption apparatus, BELSORP-18 was used for the large crystals, while BELSORP-MINI was used for the milled particles and 21-µm particles.

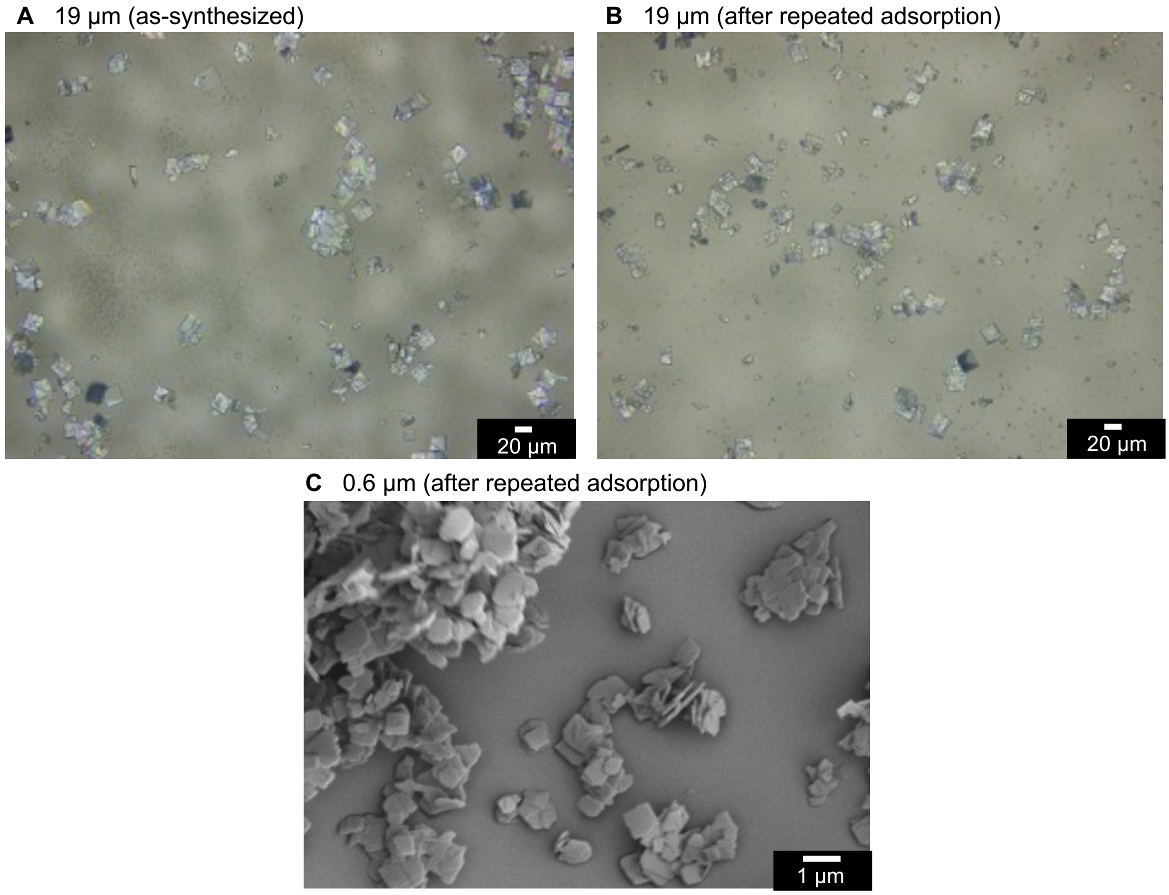

Fig. S5.

Optical microscope and SEM images of ELM-12 particles used in the repeated adsorption measurements. (**A**) As-synthesized 19-µm particles. (**B**) 19-µm particles after the adsorption measurements shown in Fig. 6C. (**C**) 0.6-µm particles after the adsorption measurements shown in Fig. 6D. The as-synthesized 0.6-µm particles correspond to Fig. S2A.

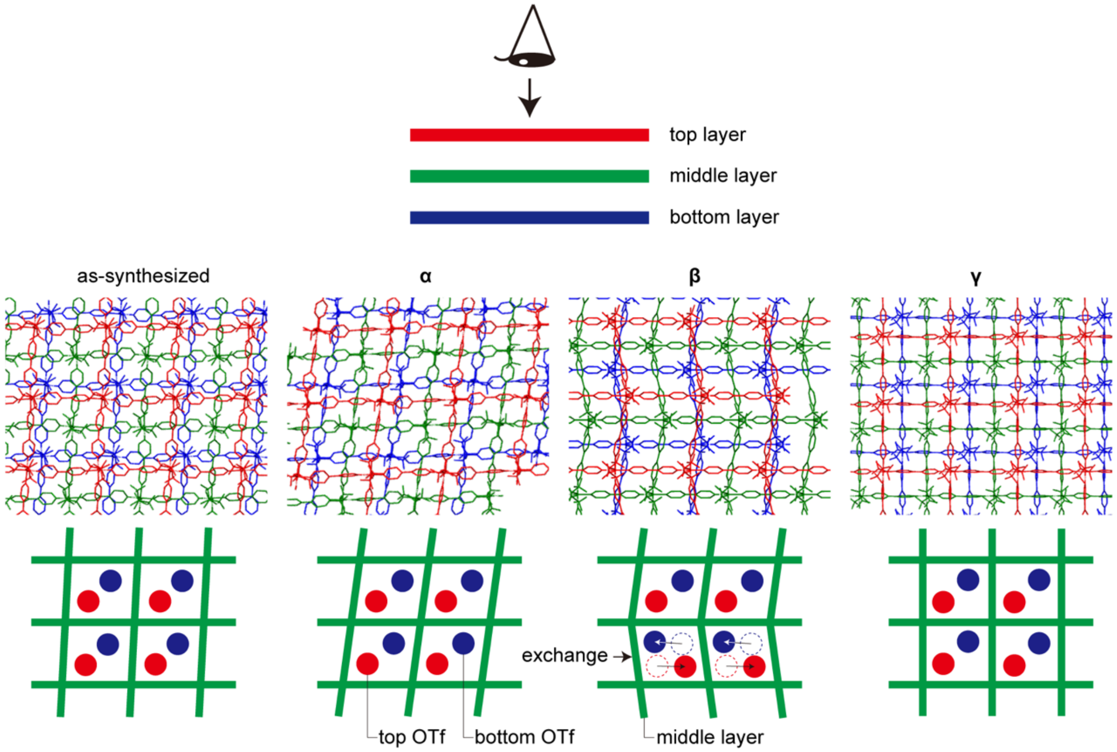

Fig. S6.

Schematic illustration of the framework structure of ELM-12. The exchange of relative positions of OTf anions is required for the structural transition between phase **β** and other structures.

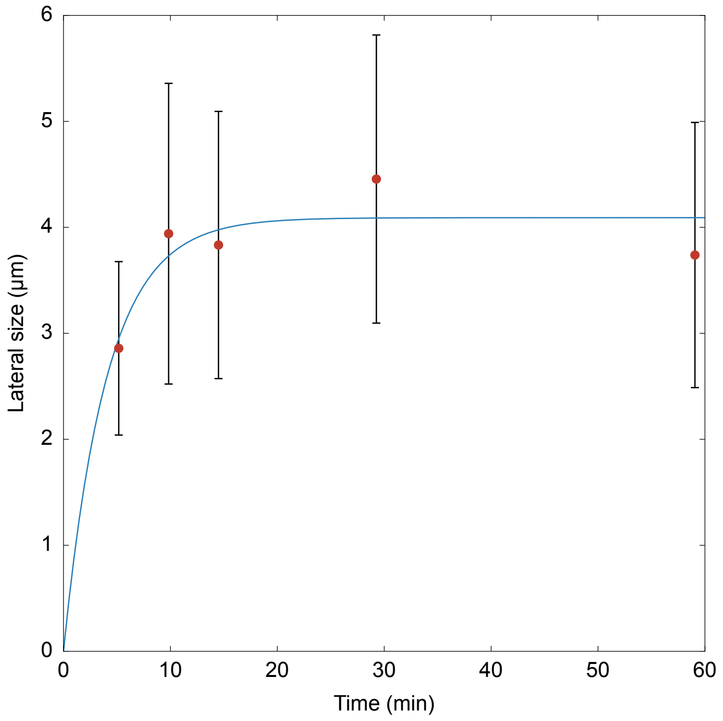

Fig. S7.

Time course of the lateral size of synthesized ELM-12 at a temperature of 10 ºC, with a Cu concentration of 30 mM and a Cu-to-bpy ratio of 1:2. Error bars indicate standard deviation of data, and the blue line represents the regression line of $D = -k_1\{\exp(-k_2 t) - 1\}$, where D is the lateral size, t is the time, $k_1 = 4.09$ µm, and $k_2 = 0.247$ min^{-1}.

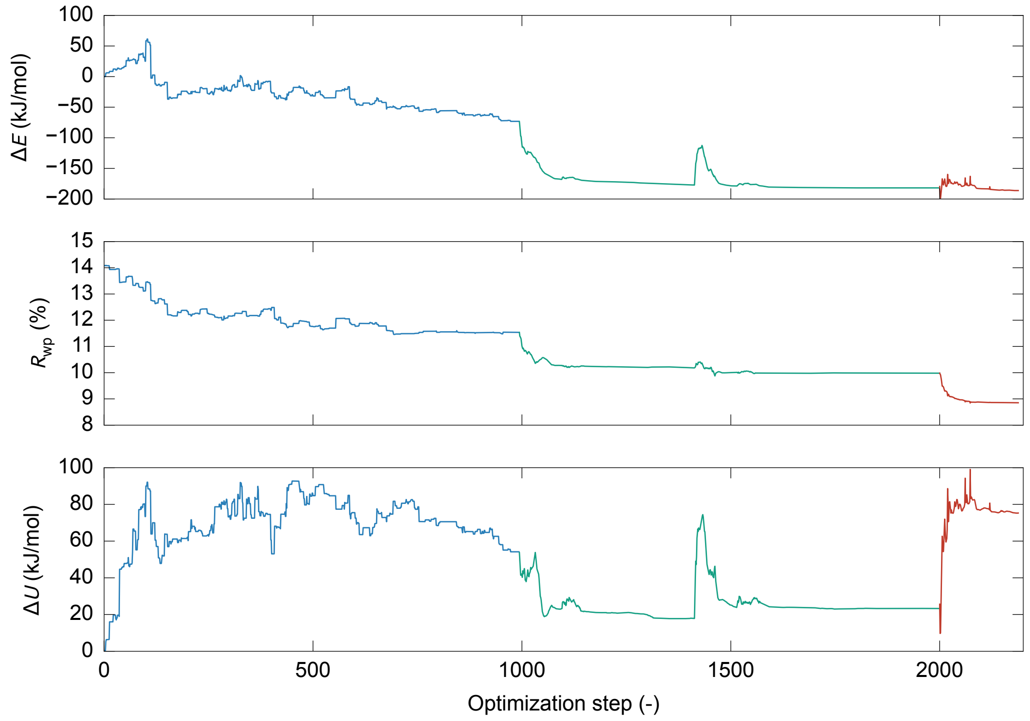

Fig. S8.

Developments of ΔE, R_{wp}, and ΔU during our structural refinement method for γ. Blue, green, and red lines represent MC simulation, molecule-level minimization, and atomic-level minimization, respectively.

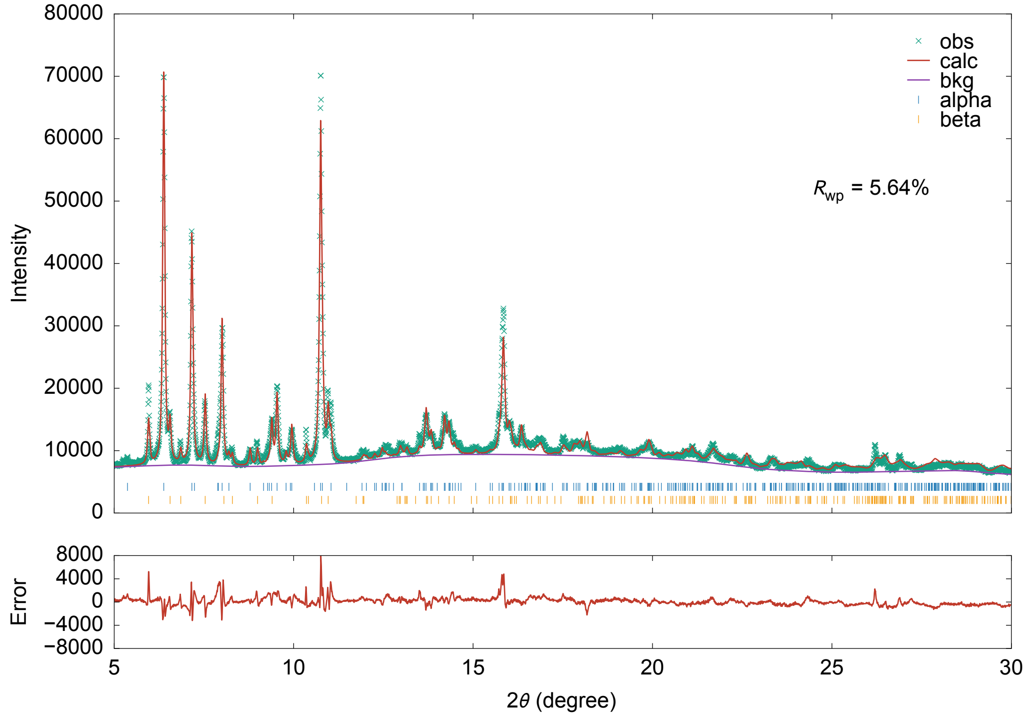

Fig. S9.

Final Rietveld fitting result for α.

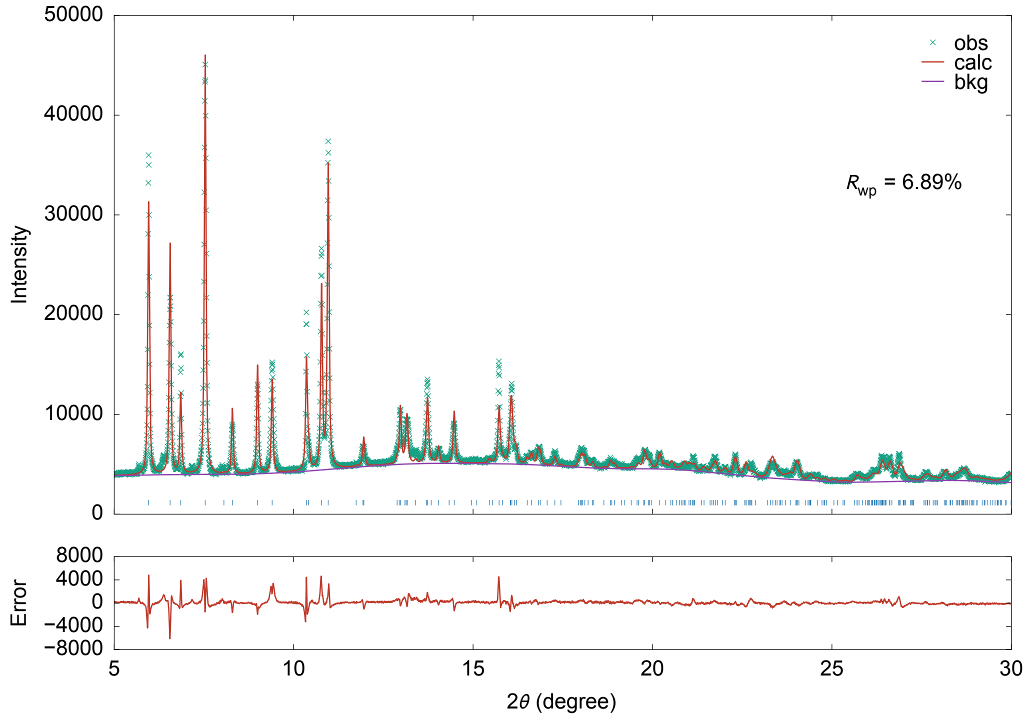

Fig. S10.

Final Rietveld fitting result for β.

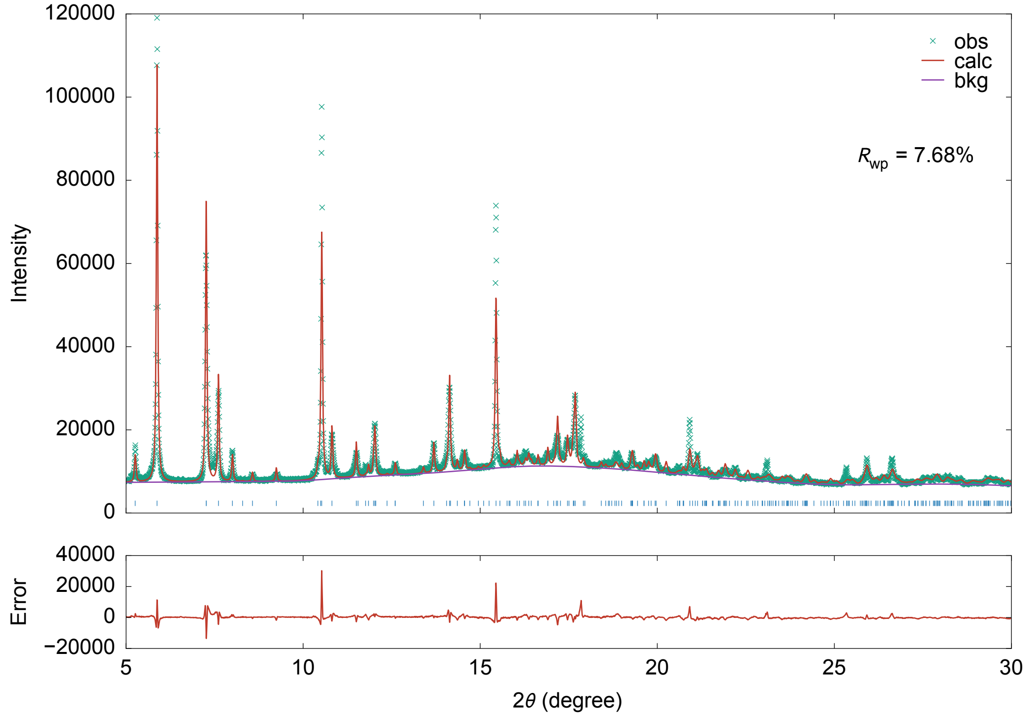

Fig. S11.
Final Rietveld fitting result for γ.
